# Muscarinic Receptor Type-3 in Hypertension and Cholinergic-Adrenergic Crosstalk: Genetic Insights and Potential for New Antihypertensive Targets

**DOI:** 10.1016/j.cjca.2019.02.003

**Published:** 2019-05

**Authors:** Rhéure Alves-Lopes, Karla B. Neves, Rhian M. Touyz

**Affiliations:** Institute of Cardiovascular and Medical Sciences, University of Glasgow, Glasgow, Scotland

Hypertension is a major risk factor for heart disease, renal failure, stroke, and vascular dementia and is the single most important cause of mortality worldwide.^1^ High blood pressure is easily detected and many effective and inexpensive antihypertensive drugs are available. It is a reversible condition and when appropriately treated, cardiovascular events are significantly reduced. However, hypertension remains a major challenge globally, with 30%-35% of adults having hypertension of whom less than 30% are treated and of those only a small proportion (10%-20%) are adequately controlled.^2^ Numerous factors contribute to these disappointing statistics including the fact that exact mechanisms that cause hypertension remain elusive. Blood pressure is regulated by complex interacting physiological systems (cardiac, vascular, renal, endocrine, neural, and immune), which are influenced by many environmental factors.^3^ In addition, multiple genes within each system, epigenetics, and epistasis (gene–environment interactions) contribute to blood pressure control.^4^ Hypertension and blood pressure are polygenic traits and genome-wide association studies have defined hundreds of localizing quantitative trait loci (QTLs) and single-nucleotide polymorphisms associated with blood pressure and hypertension.^4^ Validating each genomic marker and identifying QTL/single-nucleotide polymorphism-associated mechanistic and functional pathways in hypertension is a massive challenge, especially as the list of genomic variants associated with blood pressure and hypertension continues to grow.

In the current issue of the *Canadian Journal of Cardiology*, Deng et al.^5^ focus on unravelling potential mechanisms of the blood pressure effects of *Chrm3*, which encodes the muscarinic cholinergic receptor 3 (M3R). The basis for this relates to findings from their previous study in which they identified a novel QTL containing *ChrmM3* that contributes to salt-sensitive hypertension.^6^ Using congenic knock-ins, gene-specific knockouts and *ex vivo* and *in vivo* functional studies in the Dahl salt-sensitive rat model of polygenic hypertension, Deng et al. showed a lone missense mutation (T1776C) in the last intracellular domain of *Chrm3*, which was related to an increase in blood pressure.^5^, ^6^ Moreover, this mutation was associated with increased M3R signalling and adrenal production of epinephrine.^5^ Although these findings do not prove that the *Chrm3* mutation causes hypertension they highlight novel prohypertensive mechanisms and pathways involving cholinergic signalling and adrenal epinephrogenesis through M3R.

The cholinergic system plays an important role in regulating vascular tone, by stimulating production of vasoactive factors including nitric oxide (NO), a potent endothelium-derived vasodilator. Cholinergic transmission involves release of the neurotransmitter acetylcholine (ACh) followed by activation of postsynaptic receptors of which 2 types of ACh receptors have been identified: muscarinic and nicotinic.^7^ Mammals possess 5 distinct subtypes of muscarinic receptors, M1R-M5R, which are G-protein coupled receptors that mediate distinct responses via second messengers in different tissues and organs. In the cardiovascular system, vessels possess multiple muscarinic receptor subtypes, including M1R, M2R, M3R, and M5R, whereas the heart expresses predominantly M2Rs.^7^, ^8^, ^9^ Cardiac muscarinic stimulation causes slowing of heart rate through direct G protein-dependent regulation of ion channel activity and modulation of cyclic adenosine monophosphate (cAMP)-mediated responses.^8^ Ach-induced stimulation of muscarinic receptors in arteries causes contraction and relaxation depending on the vascular bed and the integrity of the endothelium.^7^ The primary muscarinic response in intact vessels is vasodilation caused by endothelial M3Rs (as well as M2Rs and M5Rs), which increase endothelial nitric oxide synthase (eNOS)-induced production of NO.^7^, ^10^ M3Rs seem to be particularly important because pharmacological stimulation of M3Rs with Compound 1213, an M3R-selective ligand, was shown to induce vasodilation and reduce blood pressure in experimental models of pulmonary hypertension.^11^ In the absence of endothelium or in conditions associated with endothelial injury, which is common in cardiovascular disease, ACh causes vasoconstriction by stimulating vascular smooth muscle cell M1R and M3R, processes associated with increased vascular tone and blood pressure elevation.^12^, ^13^, ^14^

Beyond the direct vascular effects of ACh-stimulated M3Rs, the cholinergic system regulates blood pressure through adrenal-derived hormones and vasoactive factors, which are also important in hypertension.^15^ As highlighted in this issue of the *Canadian Journal of Cardiology*, M3R-sensitive mechanisms stimulate adrenal production of epinephrine, which in turn influences vascular function in salt-sensitive hypertension (Fig. 1).^5^ Epinephrine induces effects through multiple α and β adrenergic receptors (adrenoceptors) in the cardiovascular system.^15^, ^16^ In the heart, epinephrine acts predominantly via activation of β1 receptors causing positive chronotropic, inotropic, and dromotropic effects. In vascular smooth muscle cells epinephrine responses involve α- and β-adrenoreceptors, causing vasoconstriction and vasodilation, respectively, with the net functional response determined by the expression profiles of adrenergic receptor subtypes. Vascular effects are largely regulated by α1 adrenergic receptors, which mediate smooth muscle contraction by mechanisms involving phospholipase C and ionositol trisphosphate-dependent increases in intracellular Ca^2+^, which lead to increased vasoreactivity and vascular tone, critically important in the pathophysiology of hypertension.^17^ Activation of smooth muscle β_2_ receptors causes relaxation, especially important in bronchi and veins.^15^, ^16^ Although epinephrine and norepinephrine directly influence vascular function, Deng et al. suggest that catecholamine-dependent hypertension through M3R is in large part through the adrenal-epinephrine-vascular axis and might be dissociated from direct M3R-induced vascular effects.^5^

**Figure 1:**
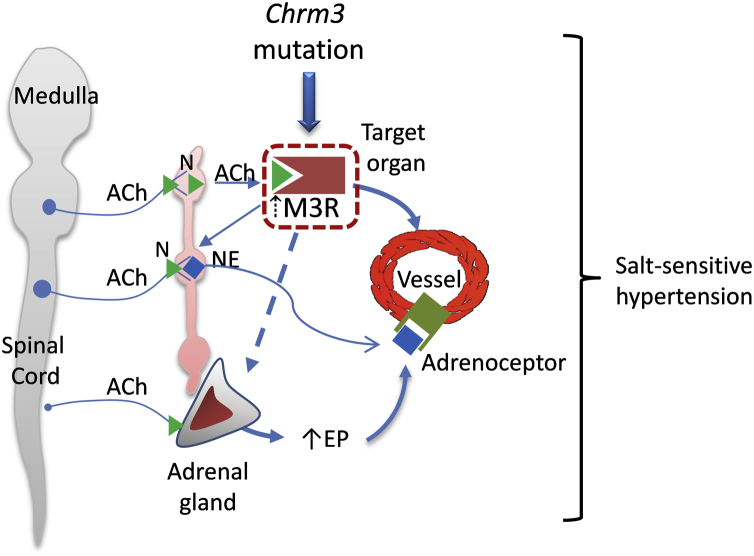
Potential mechanisms whereby a missense mutation in *Chrm3* promotes increased cholinergic signalling and stimulation of adrenal gland production of EP, which signals through vascular adrenoceptors. In vascular smooth muscle cells, *Chrm3* receptor mutation causes increased vascular contraction and tone and reduced vasodilation leading to increased blood pressure in salt-sensitive models. Muscarinic cholinergic receptor 3 (M3R) is expressed in multiple tissues and organs represented as “target organs” in the illustration. Thickness of arrow lines indicates predominant pathway. ACh, acetylcholine; N, nicotinic receptor; NE, norepinephrine; EP, epinephrine.

The concept of pharmacologically targeting muscarinic receptors to modulate norepinephrine release and vascular function was suggested more than 50 years ago when effects of nicotinic drugs were tested on peripheral adrenergic nerves and cardiac and vascular function.^18^ The novel pathway of cholinergic-adrenergic cross-talk described in this issue^5^ of the *Canadian Journal of Cardiology* highlights M3R as a putative therapeutic target in hypertension, especially in the context of *Chrm3* mutations, increased M3R signalling, and adrenal epinephrogenesis. Corroborating the findings of Deng et al.,^5^ early studies showed that muscarinic antagonists inhibit norepinephrine release evoked by ACh in rabbit, guinea pig, and cat hearts and in canine saphenous vein.^19^, ^20^, ^21^ Additionally, nicotinic cholinergic receptors on perivascular adrenergic nerves in cerebral vessels inhibit ACh-stimulated norepinephrine release.^21^

Blood pressure regulation and development of hypertension through interplay between cholinergic and adrenergic transmission via muscarinic receptors is an interesting notion that provides a platform for the integration of multiple physiological systems (vascular, cardiac, endocrine, central nervous system). However, the muscarinic-adrenergic axis is very complex because multiple muscarinic and adrenergic receptor subtypes are involved, receptors are expressed in a tissue-specific manner, and vascular functional responses depend on the integrity of the endothelium. In this issue *Canadian Journal of Cardiology*, Deng et al.^5^ unravel some of these complexities and suggest that cholinergic M3R signalling through adrenergic mechanisms involving adrenal-derived epinephrine is a novel pathway underlying hypertension. Although this is a plausible paradigm, there are some limitations that warrant consideration. First, the experiments were conducted in a salt-sensitive model (Dahl salt-sensitive rats) and it is unclear whether a similar M3R-dependent mechanism might be functionally important in salt-independent hypertension. Second, the study used genetic manipulation of *Chrm3* in rats. Whether *CHRM3* mutations in humans also exhibit cholinergic-adrenergic-dependent blood pressure associations is unclear. Third, there was enormous variability in adrenal production of epinephrine when *Chrm3* was downregulated/knocked out. Finally, the lone missense T1776C mutation of *Chrm3* was not shown to directly increase blood pressure in Dahl-sensitive rats. Despite these shortcomings, targeting M3R to reduce catecholamine levels and to downregulate the adrenergic system might be an interesting strategy to treat hypertension. In support of this beneficial metabolic effects targeting M3R in transgenic mice have been shown,^22^, ^23^ and the M3R ligand Compound 1213 reduced systemic and pulmonary arterial blood pressure in models of pulmonary hypertension.^11^ However, it should be stressed that muscarinic receptors play a critical role in regulating endothelial NO release and accordingly there is a risk of compromising vascular relaxation when muscarinic receptors are blocked. Hence, although modulation of M3R might indeed be an attractive therapeutic target in hypertension, balancing the vasodilator and vasoconstrictor effects will require careful consideration and further investigation.

## Funding Sources

R.M.T. is supported by a British Heart Foundation Chair award (CH/4/29762). K.N. and R.A.-L. are supported by the British Heart Foundation Award of Research Excellence (RE/13/5/30177).

## Disclosures

The authors have no conflicts of interest to disclose.
